# 

**Published:** 1887-01-22

**Authors:** 


### THE HOSPITAL

An Institution, Family, and Parochial Journal of
Hospitals, Asylums, & all Agencies for the Care of the Sick, Criticism & News.

### CONTENTS.

**From the Home to the Hospital** - - - 275

**Words of Consolation.**—XVII. Sympathy - - 276

**The After-Care of Asylum Convalescents.**—An Interview with Lord Brabazon - - - 277

**Hospital Funds and the Working Classes.** By Alderman F. R. Spark - - - - 278

**The Editor's Letter Box.**—

The Peoples' Want in North London - - - 280

The Wesleyans and Hospital Sunday - - - 280

Hospitals, Schools, and Boys and Girls - - - 280

**A Noble Profession: Nursing the Sick.**—Nursing in America. By Miss Alice Fisher, Matron of the Philadelphia Hospital - - - - 281

**Homes for Incurables:** Free Payments *v.* the Voting System. By Miss Louisa Twining - - - 281

**Notes and News** - - - - - - 282

**Annotations.**—Is Alcohol a Food?—Word Puzzles and Gambling.—A Royal Commission on Hospitals - - 285

**Difficult Cases** - - - - - - - 285

**Metropolitan Poor-law Infirmaries and Medical Teaching.** By Lieut.-Col. Montefiore 286

**The Common Accidents of Every-day Life.** By A. W. Hare, M.B., F.R.C.S.E.—Early Misadventures; the Infant; the Dangers Connected with Swallowing; Causes of Choking: their Symptoms and Treatment - - - - - - - - 288

**Books, Good and Otherwise** - - - 289

**Incidents of Animal Life.** Collated by the Rev. F. O. Morris, B.A.—Stories about Dogs - - 290

**The Prize Competitor** - - - - - 290

**The Children's Corner.**—Puzzles, Answers, etc. - 291

**In- and Out-Patients' Hospital Diary** - - 292

